# Keratin 23 promotes telomerase reverse transcriptase expression and human colorectal cancer growth

**DOI:** 10.1038/cddis.2017.339

**Published:** 2017-07-27

**Authors:** Ningning Zhang, Rui Zhang, Kun Zou, Wendan Yu, Wei Guo, Yingying Gao, Jia Li, Mei Li, Yidi Tai, Wenlin Huang, Chun Song, Wuguo Deng, Xiaonan Cui

**Affiliations:** 1 The First Affiliated Hospital and Institute of Cancer Stem Cell, Dalian Medical University, Dalian, China; 2 Sun Yat-sen University Cancer Center, State Key Laboratory of Oncology in South China, Collaborative Innovation Center for Cancer Medicine, Guangzhou, China; 3 Department of Colorectal Surgery, Cancer Hospital of China Medical University, Liaoning Cancer Hospital and Institute, Shengyang, China; 4 State Key Laboratory of Targeted Drug for Tumors of Guangdong Province, Guangzhou Double Bioproduct Inc, Guangzhou, China; 5 Shanghai East Hospital, Tongji University, Shanghai, China

## Abstract

The overexpression of human telomerase reverse transcriptase (hTERT) has been associated with the proliferation and migration of colorectal cancer (CRC) cells. We investigated the roles of KRT23 and hTERT in promoting CRC cell proliferation and migration. We verified the relationship between KRT23 and hTERT in CRC using streptavidin-agarose pulldown and chromatin immunoprecipitation (ChIP) assays. One hundred and fifty-four human CRC specimens were analyzed using immunohistochemistry. The roles of KRT23 and hTERT in cell growth and migration were studied using siRNA and lentiviruses *in vivo* and *in vitro*. Western blot and wound scratch analyses were used to determine the signaling pathway for KRT23-mediated activation of CRC growth and migration. Telomerase activity was measured by using the TeloTAGGG Telomerase PCR ELISA PLUS Kit. We identified KRT23 as a new hTERT promoter-binding protein. Patients with high KRT23 and hTERT expression had markedly shorter overall survival. Overexpression of KRT23 upregulated the expression of hTERT protein, hTERT promoter-driven luciferase and telomerase activity in CRC. Conversely, inhibition of KRT23 by a KRT23-specific siRNA repressed the endogenous hTERT protein, the expression of hTERT promoter-driven luciferase and telomerase activity. Overexpression of KRT23 also promoted CRC proliferation and migration. By contrast, KRT23 inhibition significantly inhibited tumor cell growth *in vitro* and *in vivo*. KRT23 promoted cancer stem cell properties and increased the expression of CD133 and CD44. These results demonstrate that KRT23 is an important cellular factor that promotes CRC growth by activating hTERT expression and that KRT23 is a potential novel therapeutic target for CRC.

Colorectal cancer (CRC) is one of the most common malignancies and the third most common cause of cancer-related mortality in the world.^1^ Additionally, CRC is becoming the fastest growing cancer in our society.^2^

It has been studied that most of the colon cancers were started from polyps. If polyps are detected early and removed, it can prevent the CRC. Therefore, it is greatly important to identify CRC at an early stage. Recent studies have reported that a few molecular biomarkers can detect CRC at the early stage and also the progression of the diseases, but there is still a huge gap that needs to be filled to improve the screening, prevention and treatment of CRC. Over the past 20 years, there are several screening strategies for detection of CRC, but it has been proven that they usually lack proper sensitivity and specificity to screen CRC.^3^ Therefore, it is deeply required to study the molecular and genetic aspects for finding out more reliable and novel biomarkers for the early detection of CRC.

Telomerase, which is a nuclear ribonucleoprotein enzyme complex, carries its own templates and maintains its telomere length by synthesizing telomeric DNA repeats. The activity of telomerase may be linked to the processes governing cellular immortalization and senescence.^4, 5^ Telomerase activity is very high in human cancer cells, but it is absent in most normal human cells.^6^ Research has demonstrated that colorectal adenocarcinoma exhibits high levels of telomerase activity^7, 8^ and human telomerase reverse transcriptase (hTERT) has been identified as a potential biomarker for CRC.^9^ Massive cell death results when telomerase is inhibited in actively dividing tumor cells;^10^ however, the regulatory processes governing the activation and expression levels of telomerase remain areas of active research.

Keratins are the intermediate filament forming proteins of epithelial cells. Thus far, there are 54 known mammalian keratins, 28 type I (acidic, KRT9-23) keratins and 26 type II (basic-to-neutral, KRT1-8) keratins.^11^ Keratin activity is involved in cancer cell growth, invasion, metastasis and treatment responsiveness. The role of keratins as multifunctional regulators of epithelial tumorigenesis merits further exploration.^12^ KRT23 transcripts were highly induced in AsPC-1 cells (a human pancreatic cancer cell line) following treatment with a histone deacetylase inhibitor,^13^ and KRT23 is considered a tumor-associated serum antigen in patients with hepatocellular carcinoma.^14^

Over the years, researchers have thought that cancer cells are stochastic in nature and that they originate from mutations in normal adult cells. However, cancer stem cells (CSCs) have been found to influence tumorigenesis. CSCs have many characteristics that promote tumorigenesis, such as self-renewal, differentiation, multidrug resistance and metastatic potential.^15, 16, 17, 18, 19, 20^ CD44 and CD133 are well known as stem cell biomarkers that are expressed in CRC.^21, 22^ Elevated CD133 expression is correlated with tumorigenesis, metastasis and poor prognosis.^22^ CD133 is an important potential target of drug therapies because many colon cancer cell lines express CD133.^23, 24^ CD44 can promote cell–cell adhesion and maintain cell–matrix interactions.^22^ When CD44 expression is upregulated, the growth and antiapoptotic properties of tumor cells are increased.^24^

In this study, we used a biotin–streptavidin pulldown assay to seek and identify unknown and essential transcriptional regulators for hTERT, and we found that KRT23 bound to the hTERT promoter, activates hTERT transcription and increased hTERT expression. Additionally, we studied the clinical association between KRT23 and hTERT in CRC tissue samples.

## Results

### Identification of KRT23 as an hTERT promoter-binding protein

**Figure 1:**
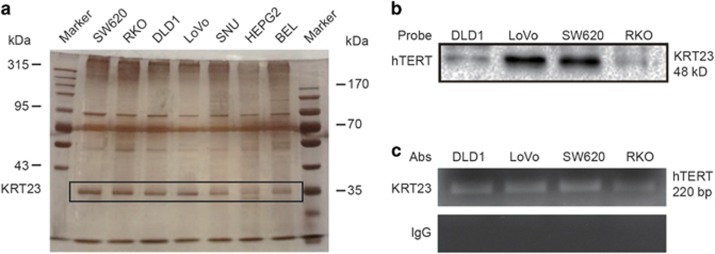
KRT23 was identified and validated as a transcription factor of hTERT in CRC cells. (**a**) A streptavidin–biotin pulldown assay was performed to identify the specific proteins that bind to the hTERT promoter. Nuclear extracts prepared from human CRC cells (SW620, RKO, DLD1 and LoVo) and hepatoma carcinoma cells (SNU, HEPG2 and BEL) were incubated with a biotin-labeled hTERT promoter probe and streptavidin–agarose beads. The DNA–protein complexes were separated by SDS-PAGE, and the protein bands were visualized by silver staining. The protein bands (at ~35 kDa) were excised from the gel and identified by the mass spectrum analysis. We predicted that the candidate CRC-specific hTERT promoter-binding protein was KRT23. (**b**) Immunoblot assay for detecting KRT23 binding to the hTERT promoter probe. KRT23 protein in the DNA–protein complexes was detected by western blot assay using an anti-KRT23 antibody. (**c**) Chromatin immunoprecipitation (CHIP) assays were performed using the hTERT promoter from human CRC cells (SW620, RKO, DLD1 and LoVo). PCR products were separated on 1% agarose gels. Normal immunoglobulin G (IgG) was the negative control for the KRT23 antibody

Streptavidin-agarose bead pulldown assay was used to detect and discover the unknown promoter-regulating factors of known target genes. In this study, we used a pulldown assay to detect novel hTERT promoter-regulating proteins in CRC cells. We synthesized a 5′-biotinylated 220-bp hTERT core promoter DNA probe^25^ and used CRC cells (SW620, RKO, DLD1 and LoVo) and hepatoma carcinoma cells (SNU, HEPG2 and BEL) for the pulldown assays. After incubating nuclear protein extracts with the hTERT promoter probe and streptavidin–agarose beads, candidate complexes were pulled down, separated by SDS-PAGE and visualized by silver staining. As shown in Figure 1a, one of the protein bands (at ~35 kDa) was predominantly present in the CRC cells. The protein bands were excised from the gel and identified by mass spectrometry. We predicted that the candidate CRC-specific hTERT promoter-binding protein was KRT23.

To verify the binding of KRT23 to the hTERT promoter region in CRC cells, we performed immunoblot analyses of the proteins eluted from the biotin-streptavidin pulldown complexes using an anti-KRT23 antibody. As shown in Figure 1b, KRT23 was clearly detected in the complexes prepared from the human CRC cell lines SW620, RKO, DLD1 and LoVo. This demonstrated that KRT23 was bound to the hTERT promoter. To further confirm KRT23 as an hTERT promoter-binding protein, we analyzed the binding of KRT23 to the hTERT promoter using a ChIP assay with a specific antibody against KRT23. Normal IgG was used as a negative control. As shown in Figure 1c, the hTERT promoter was amplified in CRC cells, indicating that KRT23 was bound to the hTERT promoter.

### KRT23 and hTERT were highly expressed in CRC tissues and CRC cells

**Figure 2:**
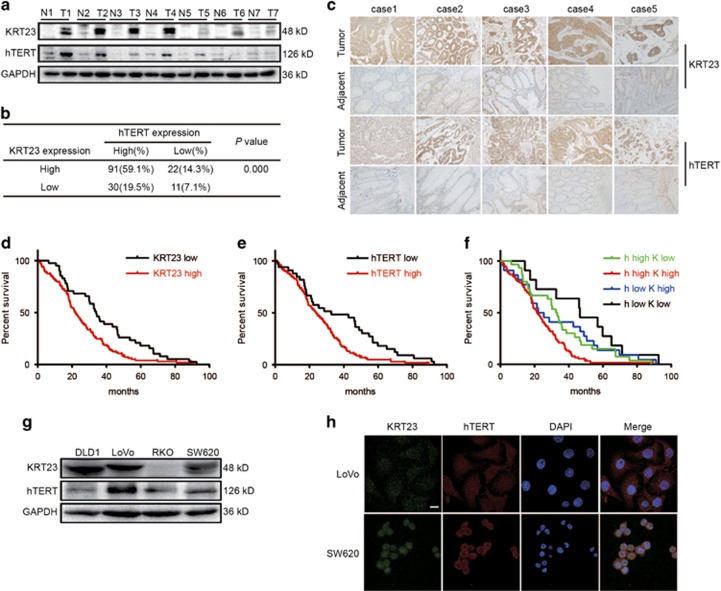
KRT23 and hTERT were highly expressed in CRC tissues and CRC cells. (**a**) Protein samples extracted from human colorectal tissues and adjacent tissues were used to evaluate the expression of KRT23 and hTERT. (**b**) The correlation between the KRT23 and hTERT expression in CRC tissues from 154 patients. (**c**) The expression and distribution of KRT23 and hTERT in CRC tissues and their adjacent non-cancer tissues through immunofluorescence analysis (× 200). (**d**) Kaplan–Meier analysis of the overall survival of CRC patients with different KRT23 expression levels (*P*<0.05, log-rank test). (**e**) Kaplan–Meier analysis of the overall survival of CRC patients with different hTERT expression levels (*P*<0.05, log-rank test). (**f**) Kaplan–Meier analysis of the overall survival of CRC patients with different KRT23 and hTERT expression levels (*P*<0.05, log-rank test). (**g**) KRT23 and hTERT expression levels in CRC cells were measured by western blot using anti-KRT23 and hTERT antibodies. (**h**) The expression levels of KRT23 and hTERT in CRC cells (LoVo and SW620) were detected by immunofluorescence assay. The localization of KRT23 and hTERT is shown. Green, KRT23; red, hTERT; blue, nuclei (× 1200)

To confirm the correlation between KRT23 and hTERT and their biological and clinicopathologic significance in CRC patients, we first measured KRT23 and hTERT expression in CRC tissues and their corresponding adjacent non-cancer tissues by western blot. Both KRT23 and hTERT were highly expressed in CRC tissues compared with their adjacent non-cancer tissues (Figure 2a). Similarly, immunohistochemical staining revealed that both KRT23 and hTERT were highly expressed in CRC tissues compared with the adjacent non-cancer tissues (Figure 2c). Of the 154 patient tumor tissue samples, ~91 (59.1%) patients had high expression of both KRT23 and hTERT (Figure 2b).

We further analyzed the effect of KRT23 and/or hTERT expression on the survival rate of 154 patients with CRC (Table 1). The overall survival curve analysis indicated that CRC patients with high KRT23 or hTERT expression had a relatively poor prognosis compared with those with weak KRT23 or hTERT expression (Figures 2d and e). Moreover, CRC patients with high expression levels of both KRT23 and hTERT had a significantly poorer prognosis (Figure 2f), which suggests that KRT23 has an important role in CRC and is associated with hTERT in predicting the prognosis of patients with CRC.

Next, we measured the expression of KRT23 and hTERT in CRC cells by western blot analysis. As shown in Figure 2g, KRT23 was highly expressed in the CRC cell lines DLD1, LoVo and SW620, but it was expressed at low levels in RKO CRC cells, whereas hTERT was expressed in all four CRC cell lines. We chose LoVo and SW620 cells to analyze the expression and subcellular localization of KRT23 and hTERT by immunofluorescence analysis. KRT23 and hTERT were both highly expressed in the nucleus in LoVo and SW620 cells (Figure 2h).

### KRT23 regulated mRNA and protein expression and luciferase activity of hTERT and telomerase activity

**Figure 3:**
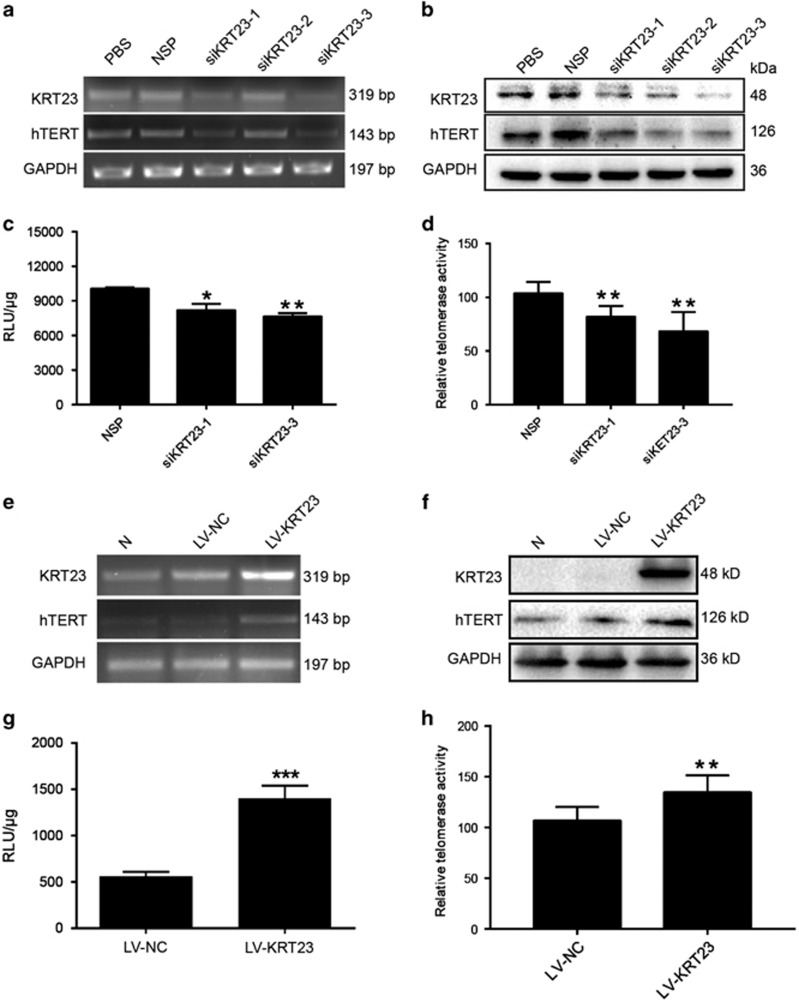
KRT23 promoted hTERT expression. (**a** and **b**) Downregulation of hTERT mRNA and protein expression by siKRT23 were analyzed by RT-PCR and western blot in LoVo cells. Luciferase activity (**c**) and telomerase activity (**d**) were measured after transfection with siKRT23-1/3 in LoVo cells. Upregulation of hTERT mRNA and protein expression by LV-KRT23 were analyzed by RT-PCR (**e**) and western blot (**f**) in RKO cells. Luciferase activity (**g**) and telomerase activity (**h**) were detected after infection of RKO cells with LV-KRT23

To further confirm the role of KRT23 in regulating hTERT expression, we evaluated the effect of KRT23 on hTERT mRNA and protein expression and luciferase and telomerase activities in LoVo cells, which express high levels of KRT23 (Figure 2g). First, we examined the effect of three different KRT23 siRNAs on the mRNA and protein expression levels of KRT23 and hTERT. The mRNA and protein expression levels of KRT23 and hTERT were both decreased in cells treated with KRT23 siRNA (Figures 3a and b). Then, we measured the effect of KRT23 on hTERT promoter-driven luciferase activity and telomerase activity using two KRT23 siRNAs with strong silencing effects. The hTERT promoter-driven luciferase and telomerase activities were significantly inhibited by both siKRT23-1 and siKRT23-3 (Figures 3c and d).

Next, we evaluated the effect of KRT23 on hTERT mRNA and protein expression and luciferase activity and telomerase activity in RKO CRC cells, which expressed low levels of KRT23 (Figure 2g). First, we examined the infection efficiency of lentiviruses for KRT23 overexpression. As shown in Figures 3e and f, the mRNA and protein expression levels of KRT23 and hTERT were both increased in cells treated with KRT23 lentivirus (the infection efficiency of the lentiviruses was almost 100% at 10^8^ TU/ml). However, the expression of KRT23 at mRNA level was greatly stronger than KRT23 at the protein level. We thought that the protein of KRT23 was degraded after its translation, like postmodification. Additionally, we measured the effect of KRT23 on hTERT promoter-driven luciferase activity using lentiviruses. The hTERT promoter-driven luciferase and telomerase activities were significantly activated by lentivirus (Figures 3g and h).

### Knockdown of KRT23 inhibited the proliferation and migration in LoVo CRC cells

**Figure 4:**
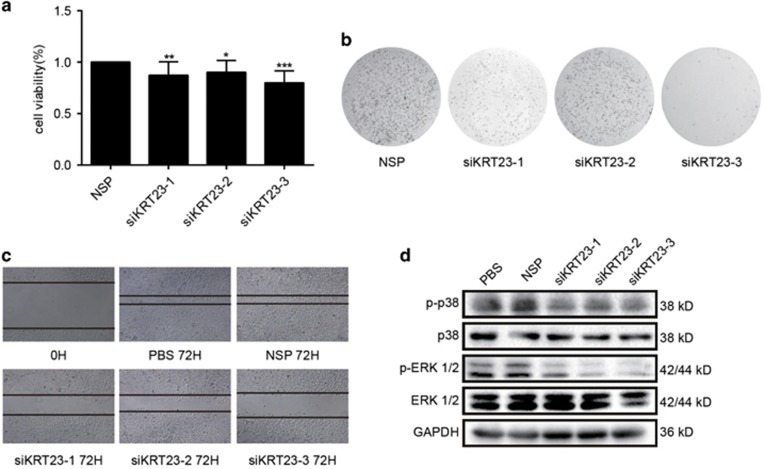
Knockdown of KRT23 inhibited the proliferative capacity and migration of LoVo CRC cells. (**a**) Cell viability was analyzed by MTT in LoVo cells that were transfected with siKRT23 or NSP for 48 h. (**b**) Colony formation assay of LoVo cells transfected with siKRT23 or NSP two times a week for 2 weeks. (**c**) Cell migration was analyzed by a wound-healing assay. LoVo cells were seeded in 6-well plates and grown to full confluence (50 ×). (**d**) The phosphorylated and total ERK and p38 levels in LoVo cells transfected with siKRT23 were detected by western blot assay using antibodies against p-p38, p38, p-ERK and ERK (*P<0.05, **P<0.01 and ***P<0.001)

Because KRT23 had a positive effect on the activation of hTERT expression, we reasoned that KRT23 might be a potential therapeutic target in CRC treatment. As a result, we sought to determine the effects of KRT23 on CRC growth. First, we examined the proliferative capacity of LoVo cells after using siRNA to inhibit KRT23 expression. Inhibition of KRT23 expression with siKRT23 markedly suppressed cell proliferation compared with nonspecific control siRNA (NSP) in LoVo cells (Figure 4a). Then, we examined the colony formation and migration of LoVo cells after using siRNA to inhibit KRT23 expression. The results showed that similar to proliferation, the colony formation and migration of LoVo cells were inhibited by siKRT23 (Figures 4b and c). The activation of extracellular signal-regulated protein kinase 1/2 (ERK1/2) and p38 is mainly associated with cell survival, proliferation, migration and cell growth. We measured MAPK family protein expression and verified the role of KRT23 in activating the ERK1/2 and p38 MAPK proteins. As shown in Figure 4d, the phosphorylation of ERK1/2 and p38 was inhibited by siKRT23, but the total ERK1/2 and p38 levels were not changed. From these results, we conclude that KRT23 is correlated with cell proliferation and migration through regulating the ERK1/2 and p38 signaling pathways.

### Overexpression of KRT23 promoted the proliferation and migration of RKO CRC cells by p38 and ERK 1/2 signaling pathway

**Figure 5:**
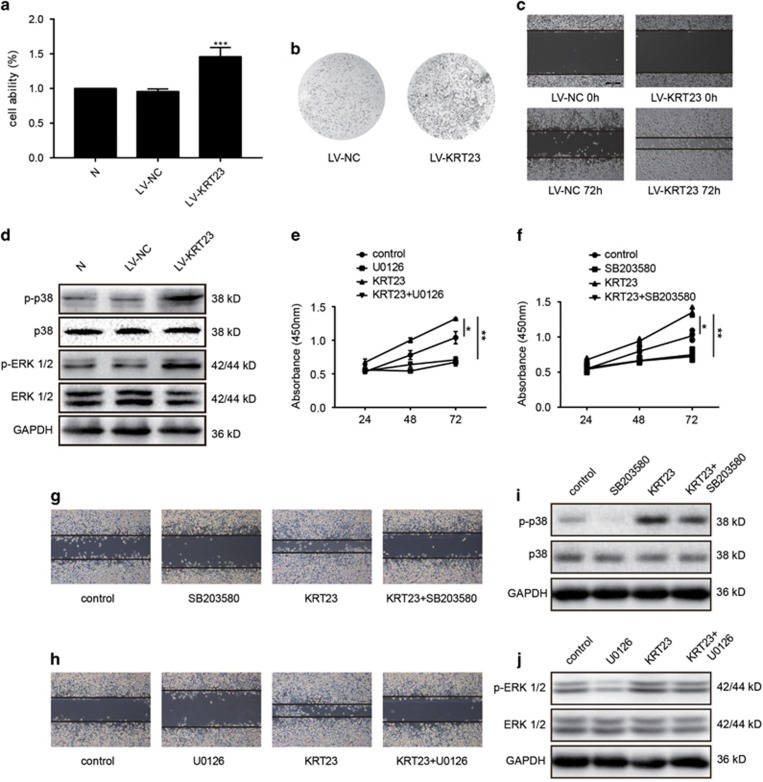
Overexpression of KRT23 promoted proliferation and migration in RKO CRC cells. (**a**) Cell viability was analyzed by MTT in RKO cells that were infected with or the NSP LV-NC. (**b**) Colony formation assay of RKO cells infected with LV-KRT23 or the NSP LV-NC. (**c**) Cell migration was analyzed by a wound-healing assay. RKO cells were seeded in 6-well plates and grown to full confluence (50 ×). (**d**) The phosphorylated and total ERK and p38 levels in RKO cells infected with LV-KRT23 were measured by a western blot assay using antibodies against p-p38, p38, p-ERK and ERK. (**e**) Cell viability was analyzed by CCK-8 in RKO cells that were treated with U0126 and/or LV-KRT23. (**f**) Cell viability was analyzed by CCK-8 in RKO cells that were treated with SB203580 and/or LV-KRT23. (**g**) Cell migration was analyzed by a wound-healing assay. RKO cells were treated with SB203580 and/or LV-KRT23. (**h**) Cell migration was analyzed by a wound-healing assay. RKO cells were treated with U0126 and/or LV-KRT23. (**i**) The phosphorylated and total p38 levels in RKO cells treated with SB203580 and/or LV-KRT23 were measured by a western blot assay using antibodies against p-p38 and p38. (**j**) The phosphorylated and total ERK 1/2 levels in RKO cells treated with U0126 and/or LV-KRT23 were measured by a western blot assay using antibodies against p-ERK 1/2 and ERK 1/2 (*P<0.05, **P<0.01 and ***P<0.001)

We observed that KRT23 knockdown inhibited the proliferation and migration of LoVo CRC cells. In this part, we detected the effect of overexpressed KRT23 on RKO CRC cells. First, we examined the proliferative capacity of RKO cells after using a lentivirus to promote KRT23 overexpression. The results showed that the lentivirus-mediated overexpression of KRT23 markedly increased the cell proliferation of RKO cells compared with the NSP (Figure 5a). Then, we examined the colony formation and migration of RKO cells after using a lentivirus to promote KRT23 overexpression. The results showed that similar to the proliferation, the colony formation activity and migration of RKO cells were increased by KRT23 lentivirus infection (Figures 5b and c). Additionally, we measured the MAPK family protein expression and verified the role of KRT23 in activating the ERK1/2 and p38 MAPK proteins. As shown in Figure 5d, the phosphorylation of ERK1/2 and p38 was increased by lentivirus treatment, but the total ERK1/2 and p38 levels were not changed. Then, RKO cells were divided into four groups as follows: (i) the control group; (ii) the SB203580-treated group (the inhibitor of p38) or U0126-treated group (the inhibitor of ERK 1/2); (iii) the KRT23 lentivirus-treated group; (iv) the SB203580+ KRT23 lentivirus-treated group or U0126+ KRT23 lentivirus-treated group. We used CCK-8 assay to detect the ability of proliferation and wound assay to detect the ability of migration in these four groups. The results showed that inhibitors could reverse the ability of cell proliferation (Figures 5e and f) and migration (Figures 5g and h), which was promoted by KRT23 lentivirus. Additionally, we measured the expression of p-ERK1/2 and p-p38 MAPK. As shown in Figures 5i and j, the inhibitors could reverse the phosphorylation of ERK1/2 and p38, which was promoted by KRT23 lentivirus, but the total ERK1/2 and p38 levels were not changed. From the results above, we again conclude that KRT23 is correlated with cell proliferation and migration by regulating the ERK1/2 and p38 signaling pathways.

### KRT23 promoted CSC properties and increased the expression of CD133 and CD44

**Figure 6:**
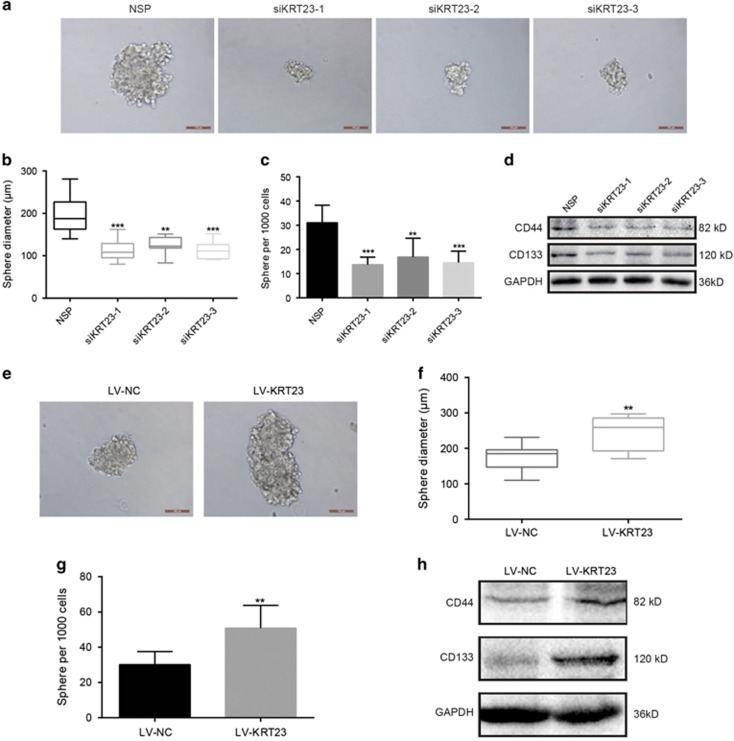
KRT23 promoted CSC properties and increased CD133 and CD44 expression levels. (**a**) Representative images of tumor cell spheres formed by LoVo cells after transfection with siKRT23-1, 2, and 3 for 2 weeks, respectively (scale bars, 100 *μ*m). (**b** and **c**) Diagrams show the diameter and number of tumor cell spheres (>50 *μ*m). (**d**) The CD44 and CD133 protein levels in the cell lysates were measured by western blot analysis after transfection with siKRT23-1, 2 and 3. (**e**) Representative images of tumor cell spheres formed by RKO cells after infection with LV-KRT23 for 2 weeks (scale bars, 100 *μ*m). (**f** and **g**) Diagrams showing the diameter and number of tumor cell spheres (>50 *μ*m). (**h**) The CD44 and CD133 protein levels in the cell lysates were measured by western blot analysis after infection with LV-KRT23 (**P<0.01 and ***P<0.001)

We chose the LoVo and RKO CRC cell lines to detect the potential role of KRT23 in promoting CSC properties. KRT23 significantly decreased the sphere number and size after siKRT23-1, -2 and -3 transfection compared with the NSP in LoVo cells (Figures 6a–c). Then, we examined the protein expression of CD44 and CD133 by western blot. Similar results were observed for the CD44 and CD133 expression levels, which were decreased after siKRT23-1, -2 and 3 transfection (Figure 6d). Additionally, we found that the sphere size and number and the protein expression levels of CD44 and CD133 after LV-KRT23 infection were significantly increased compared with LV-NC in RKO cells (Figures 6e–h). These results demonstrate that KRT23 influences the sphere-forming ability of tumors.

### Inhibition of tumor growth by KRT23 knockdown in mice

**Figure 7:**
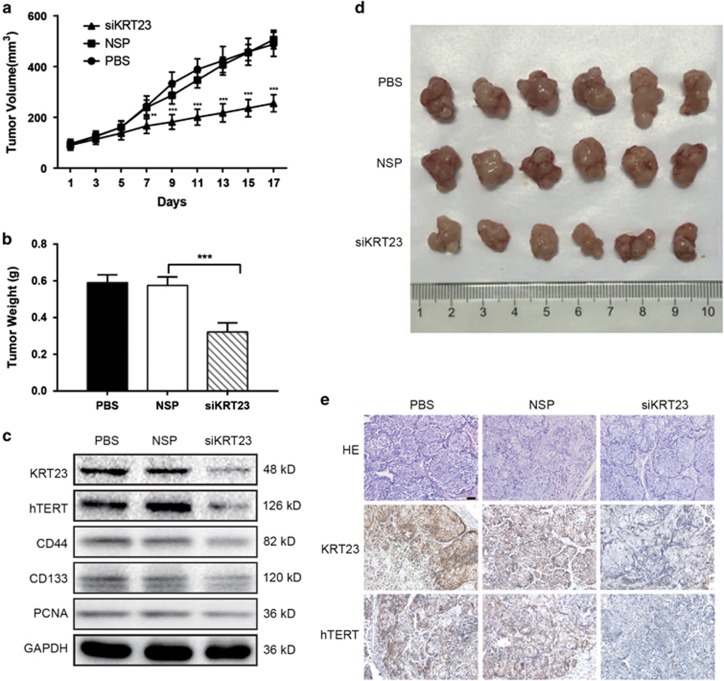
Inhibition of tumor growth by KRT23 knockdown in mice. (**a**) The tumor volume of each group of nude mice was measured and calculated as *V*=(width^2^ × length)/2, with *n*=6 and **P<0.01, ***P<0.001. (**b**) The weight of tumor grafts 17 days after the intratumoral injection of PBS, NSP or siKRT23-3, ***P<0.001. (**c**) The proteins were extracted from tumor xenografts. The KRT23, hTERT, CD44, CD133, PCNA, GAPDH expression levels were measured by western blot analysis. (**d**) The morphology of the tumor xenografts in each nude mouse after 17 days of treatment. (**e**) An immunohistochemistry assay of KRT23 and hTERT expression in tumor xenografts from each group of nude mice (× 200)

The effect of KRT23 on tumor proliferation was further examined in nude mice with human colorectal tumor xenografts. LoVo cells were subcutaneously injected into the armpits of nude mice. When the tumors were ~100 mm^3^ in size, the animals were treated with siKRT23-3 (most efficient) and NSP. As shown in Figures 7a, b and d, the tumor volume, weight and size in mice treated with siKRT23-3 were smaller and lighter than in the group treated with the NSP. Furthermore, we examined the effect of KRT23 knockdown on the protein expression of hTERT, CD44, CD133 and PCNA in xenografts by western blot and immunohistochemistry analyses. As shown in Figures 7c and e, siKRT23-3 treatment attenuated hTERT, CD44, CD133 and PCNA expression in xenografts. These results demonstrate the important role of KRT23 in CRC growth through partially regulating hTERT expression.

## Discussion

CRC is one of the most common causes of cancer death.^26^ Increasing numbers of studies have demonstrated the strong correlation between elevated hTERT expression and activation and CRC, and hTERT is thought to be a potential therapeutic target for cancer therapy. Therefore, the molecular mechanisms of hTERT in CRC must be identified. Additionally, it is known that overactivation of hTERT leads to telomere lengthening and cell immortalization, playing a crucial role in tumorigenesis and development. Conversely, degradation of hTERT by proteasomes or repression of its activity prompts telomere shortening, cancer cell senescence and apoptosis.

The expression of hTERT can be regulated by promoter methylation or chromatin remodeling,^27, 28^ as well as by activators and repressors, such as USF1/2, Sp1, TGF-*β*, p53 and c-Myc.^29, 30, 31^ This study is the first to present a novel mechanism involved in hTERT expression regulation in CRC, demonstrating that hTERT transcription activation depends on KRT23.

In this study, we focused on the molecular mechanism of transcriptional regulation underlying the observed increased expression of hTERT. We used a biotin-streptavidin pulldown assay to search for and identify the unknown and essential transcriptional regulators of hTERT. Eventually, we found that KRT23 specifically binds to the hTERT promoter, activates hTERT transcription (Figure 1) and increases hTERT expression (Figure 3). Knockdown of KRT23 also inhibited CRC cell growth *in vitro* and *in vivo* (Figures 4,5 and 7). Furthermore, the expression of KRT23 was positively correlated with that of hTERT according to an IHC assay using CRC specimens (Figure 2). We also found that KRT23 promoted CSC properties and increased the expression of CD133 and CD44 (Figure 6). All these results uncover a novel role for KRT23 as a regulator of hTERT in CRC and suggest that KRT23 can potentially be developed as an anticancer therapeutic target.

KRT23 is a member of the keratin family, which contains 50 members that are major structural proteins in epithelial cells. The keratin family can be divided into two groups, (i) acidic forms and (ii) basic forms.^32, 33^ KRT23, an acidic form, has been detected in different cancer types. Additionally, KRT23 can be used as an HCC-associated antigen in patient sera.^14^ At the molecular level, the expression of some genes (e.g., cyclin D1, cyclin E and E2F1) is decreased when KRT23 is knocked down.^13^ Additionally, KRT23 knockout colon cancer cells are restricted in their assembly of functional G1/S complexes.^34^

By analyzing the functional and structural domain of KRT23, we found that it lacks a DNA-binding domain, which is frequently needed to bind to the target gene promoter. We speculated that KRT23 might execute its coactivation effect on hTERT expression by recruiting transcription factors to bind to the hTERT promoter. Further detailed analyses are needed to determine the partner transcription factors of KRT23 during hTERT expression activation in CRC.

In summary, our findings are the first to show that KRT23 is a novel hTERT promoter-regulating protein that has an important role in hTERT overexpression and tumor growth in CRC. Our results suggest that KRT23 is a potential therapeutic target in CRC.

## Materials and Methods

### Clinical samples

All CRC tissues and CRC paraffin samples were kindly provided by the Department of Gastrointestinal Surgery, The First Affiliated Hospital of Dalian Medical University and the China Department of Colorectal Surgery, Cancer Hospital of China Medical University, Liaoning Cancer Hospital and Institute. All protocols concerning the use of patient samples in this study were approved by the Institutional Review Board of Dalian Medical University and China Medical University (Liaoning, China). A signed informed consent was obtained from each patient. The investigations were conducted according to the Declaration of Helsinki principles.

### Cell culture and transfection

SW620, RKO, LoVo and DLD1 cells were obtained from the American Type Culture Collection. SW620 and RKO cells were cultured in Dulbecco’s modified Eagle's medium (Hyclone, Logan, UT, USA.). LoVo and DLD1 cells were cultured in Roswell Park Memorial Institute (RPMI) 1640 medium (Hyclone, Logan, UT, USA.). All the cells were supplemented with 10% fetal bovine serum (Hyclone, Logan, UT, USA) and were maintained in a humidified atmosphere and 5% CO_2_ at 37 °C.

### Streptavidin-agarose pulldown assay

Transactivators binding to an hTERT core promoter probe were identified using a streptavidin-agarose pulldown assay. Briefly, a biotin-labeled, double-stranded DNA probe corresponding to nucleotides −378 to −159 bp of the hTERT promoter sequence was synthesized by Sigma (Sigma-Aldrich, St. Louis, MO, USA) (sense, 5′-ACCCTGGGAGCGCGAGCGGC-3′ and antisense, 5′-GGGGCGGGGTCCGCGCGGAG-3′). Cell nuclear protein extract (500 *μ*g), 4 *μ*g of DNA probe and 40 *μ*l of streptavidin-agarose beads (Sigma-Aldrich) were mixed and incubated at room temperature for 2 h with a rotating shaker and then pelleted by centrifugation to pull down the DNA–protein complexes. After washing with cold phosphate-buffered saline (PBS), the proteins in the complex were analyzed in further assays.

### ChIP assay

The ChIP assay was as per the manufacturer’s instructions. Briefly, the cells were fixed with 1% formaldehyde and sonicated to shear the DNA to 200 to 500 bp on ice. One-third of the total cell lysate was used as the DNA input control. The remaining two-thirds of the total lysate were subjected to immunoprecipitations with anti-KRT23 antibody or non-immune rabbit IgG. The DNA was subjected to PCR to amplify a 220 bp region (−378 to −159 bp) of the hTERT promoter using the primers (5′-ACCCTGGGAGCGCGAGCGGC-3′ and 5′-GGGGCGGGGTCCGCGCGGAG-3′). The PCR products were resolved electrophoretically on a 1% agarose gel and visualized by ethidium bromide staining.

### Reverse transcription-polymerase chain reaction

Total RNA was extracted with the RNAiso Plus (Takara, Dalian, China) reagent and quantified by the measurement of the optic absorbance at 260 and 280 nm with a resultant *A*_260_/*A*_280_ ratio that ranged from 1.8 to 2.0. The concentration of total RNA was calculated according to A260. The KRT23 primers were 5′-AATACCCGCCTGATGTCGTG-3′ (sense) and 5′-TGAAGCTGTGTCCGGAGTTC-3′ (antisense). The hTERT primers were 5′-AGGCTCACGGAGGTCATCG-3′ (sense) and 5′-GGCTGGAGGTCTGTCAAGGTA-3′ (antisense). The GAPDH primers were 5′-GGAGCGAGATCCCTCCAAAAT-3′ (sense) and 5′-GGCTGTTGTCATACTTCTCATGG-3′ (antisense). PCR reactions were performed as follows: initial denaturation at 98 °C for 3 min, 30 cycles of 98 °C for 10 s, 60 °C for 30 s and 72 °C for 1 min. The PCR reaction products were electrophoresed on 1% agarose gel, and the bands were visualized by ethidium bromide staining, followed by analysis with Labworks 4.6 (UVP, Upland, CA, USA).

### Western blotting

After extraction from cells, the protein concentration was determined with the BCA Protein Assay Kit (Beijing ComWin Biotech Co., Beijing, China). Western blotting was performed according to the manufacturer’s instructions. The proteins on the membranes were detected using ECL and visualized at Bio-Rad Laboratories (Berkeley, CA, USA). The western blots are representative of at least three independent experiments. Densitometric analysis of each band for the target protein was performed with Scion Image 4.03.

### Confocal immunofluorescence assay

Indirect immunofluorescence staining was performed according to the manufacturer’s instructions and evaluated on a Leica confocal microscope.

### Wound scratch assay

Cells were cultured to 90% confluence in six-well plates. The cells were wounded using a sterile 20 *μ*l pipette tip, and the suspended cells were washed. The scratch wounds were captured with a Leica (DMI4000B) microscope in three fields of view at × 50 magnification.

### MTT assay for cell proliferation

RKO and LoVo cell lines (2000 cells per well) seeded in 96-well plates were treated with siRNA for KRT23 or negative control siRNA, or with KRT23 lentivirus or negative control lentivirus, at the indicated doses. The cell viability was determined after 48 h of treatment.

### Telomerase activity assays

Telomerase activity was analyzed by TeloTAGGG Telomerase PCR ELISA PLUS Assay Kit (Roche Applied Science, Shanghai, China) according to the manufacturer’s instruction.

### CCK-8 assay for cell proliferation

RKO cell lines (2000 cells per well) seeded in 96-well plates were treated with SB203580 (inhibitor of p38, 1 *μ*M) or U0126 (inhibitor of ERK 1/2, 1 *μ*M) and/or KRT23 lentivirus. The cell viability was determined after 24, 48 and 72 h of treatment.

### Clone formation assay

To analyze the effect of KRT23 on the clonogenicity of tumor cells *in vitro*, RKO and LoVo cell lines (1000 cells per well) seeded in six-well plates were treated with KRT23 siRNA or negative control siRNA, or with KRT23 lentivirus or negative control lentivirus, at the indicated doses. After 14 days, the cells were washed with PBS, fixed with fixation solution (methanol: glacial acetic acid: H_2_O=1:1:8) for 10 min and stained with 0.1% crystal violet for 30 min. Clones with more than 50 cells were counted using an optical microscope.

### Sphere formation assay

Sphere formation was evaluated in ultralow-attachment plates (Corning, New York, NY, USA) with medium supplemented with 2% B27, 20 ng/ml bFGF and 20 ng/ml EGF. RKO or LoVo cells were seeded at a density of ~2000 cells/ml and cultured at 37 °C in 5% CO_2_. After 2 weeks, the spheres were counted at × 200 magnification when their diameters were >50 *μ*m. Sphere formation efficiency (SFE)=number of spheres per 1000 cells.

### Xenograft tumor models of human CRC

Animal experiments were carried out in accordance with the National Institute of Health Guide for the Care and Use of Laboratory Animals, with the approval of the Animal Research Committee of Dalian Medical University. Male nude mice were obtained from the Dalian Medical University. The animals (4–6 weeks of age, 18–25 g) were housed in a specific pathogen-free (SPF) environment and provided sterile water and food *ad libitum*. The mice were used for experiments after they had acclimatized for 1 week. LoVo cells (5 × 10^6^) that were suspended in 200 *μ*l of PBS were subcutaneously injected into the right flanks of the mice. The mice were randomly divided into three groups (six mice per group) after the tumor volume reached 100 mm^3^: (a) PBS, (b) control siRNA and (c) KRT23 siRNA. To deliver DC nanoparticle-conjugated siRNA, we injected 10 *μ*g of siRNA in 0.1 ml saline buffer intratumorally two times per week for 3 weeks. The whole body weight and tumor volume were measured every 2 days after tumor inoculation. The tumor volume was computed according to the following formula: tumor volume (mm^3^)=1/2 × *a* (tumor length) × *b*^2^ (tumor width). At the end of the experimental period, all animals were killed by cervical decapitation, the tumor tissues were excised aseptically, the weights were recorded and the samples were used for further study.

### Statistical analyses

The data are expressed as the mean±S.E.M. of three independent experiments with GraphPad Prism software (La Jolla, CA, USA). Student’s *t*-test was used to make a statistical comparison between groups. *P<0.05, **P<0.01 and ***P<0.001 were considered statistically significant.

## Publisher's Note

Springer Nature remains neutral with regard to jurisdictional claims in published maps and institutional affiliations.

## Figures and Tables

**Table 1:** Kaplan-meier analyses for prognosis and survival rate of 154 CRC patients

	**Frequency**	**MST**	**95% CI**		**Survival rate**		***χ*****^2^**	**P-value**
			**Lower**	**Upper**	**3 years**	**5 years**		
*Gender*								
Male	84	26.400±4.308	17.957	34.843	34.50%	13.50%	2.559	0.11
Female	70	23.000±2.353	18.388	27.612	24.30%	1.40%		
								
*Age (years)*								
≤60	69	25.400±3.322	18.888	31.912	33.30%	8.20%	0.005	0.945
>60	85	24.600±3.022	18.677	30.523	27.10%	7.50%		
								
*Location*								
Colon	50	20.900±2.576	15.851	25.949	32.00%	6.00%	1.638	0.201
Rectum	104	27.000±2.719	21.67	32.33	28.80%	8.60%		
								
*Size*								
≤5 cm	93	28.600±3.154	22.417	34.783	34.40%	9.80%	3.721	0.054
>5 cm	61	20.800±2.789	15.334	26.266	23.00%	4.90%		
								
*Histological type*								
Adenocarcinoma	132	25.400±2.503	20.494	30.306	31.10%	8.30%	0.792	0.373
Other	22	20.800±3.694	13.56	28.04	22.70%	4.50%		
								
*Differentiation*								
High	52	31.900±2.404	27.189	36.611	38.50%	15.40%	12.936	***0.002***
Moderate	88	22.500±2.345	17.903	27.097	27.30%	2.00%		
** **Low	14	10.300±4.677	1.133	19.467	14.00%	0.00%		
								
*T stage*								
T2+T3	113	26.400±2.780	20.951	31.849	33.60%	7.80%	2.615	0.106
T4	41	21.400±3.265	15.001	27.799	19.50%	7.30%		
								
*N stage*								
N0	56	28.600±6.174	16.499	40.701	44.60%	15.60%	10.912	***0.004***
N1	**52**	21.400±2.644	16.218	26.582	21.20%	5.80%		
N2	46	24.600±5.143	14.519	34.681	21.70%	0.00%		
								
*TNM*								
Stage II	56	28.600±6.174	16.499	40.701	44.60%	15.60%	10.461	***0.001***
Stage III	98	22.500±2.772	17.067	27.933	21.40%	3.30%		
								
*hTERT*								
Low	33	32.700±13.781	5.69	59.71	48.50%	18.20%	8.867	***0.003***
High	121	23.700±2.369	19.056	28.344	24.80%	5.00%		
								
*KRT23*								
Low	41	34.100±2.048	30.085	38.115	43.90%	18.20%	8.895	***0.003***
High	113	21.900±2.050	17.882	25.918	24.80%	4.00%		
								
*h(TERT)/K(RT23)*								
h low/K high	22	22.100±6.039	10.264	33.936	41.00%	13.60%	17.836	***0.000***
h low/K low	11	46.500±13.322	20.39	72.61	63.60%	27.30%		
h high/K low	30	32.600±2.876	26.964	38.236	36.70%	15.00%		
h high/K high	91	21.400±1.828	17.817	24.983	20.90%	1.60%		

The bold and italic means *P*-value<0.05.
